# Insights from a community-based survey on factors influencing acceptance and uptake of Paxlovid (nirmatrelvir and ritonavir) as a COVID-19 antiviral medication in Singapore

**DOI:** 10.1186/s12889-024-19687-0

**Published:** 2024-08-28

**Authors:** Sheng En Alexius Matthias Soh, Wei Ling Brenda Ong, Tun-Linn Thein, Konstadina Griva, I.-Cheng Mark Chen

**Affiliations:** 1 Infectious Disease Research and Training Office, National Centre for Infectious Diseases, 16 Jln Tan Tock Seng, Singapore, 308442 Singapore; 2 Population/Global Health, Lee Kong Chian School of Medicine, Nanyang Technological University, Singapore, 308232 Singapore; 3 National Public Health and Epidemiology Unit, National Centre for Infectious Diseases, 16 JIn Tan Tock Seng, Singapore, 308442 Singapore

**Keywords:** Antiviral awareness, Antiviral acceptability, Healthcare trust, Government communications, COVID-19 antiviral, Public confidence, Patient-provider communication, Decision-making, COVID-19 information, Health messaging, Trust-building

## Abstract

**Introduction:**

Antiviral treatment can reduce the burden of COVID-19. But utilisation can be suboptimal, even in a setting like Singapore where it is fully subsidized for those with selected medical conditions and older adults (≥ 50 years). We hence investigated the factors affecting awareness, acceptance, and initiative to request Paxlovid.

**Methods:**

We assessed the Paxlovid awareness, factors impacting its uptake in a survey conducted from August 2022 to September 2022 through the SOCRATES cohort. Multivariable logistic regression was used to investigate associations between sociodemographics, perceptions, and attitudes with the key study outcomes.

**Results:**

Among respondents to the Paxlovid survey, 54% were aware of Paxlovid. On being provided essential details about Paxlovid, 75% reported they would likely be receptive to taking it if prescribed, and 38% indicated the initiative to request for it if it was not suggested by their doctors. Factors associated with *awareness* of Paxlovid include aged 40 years old and above, higher education, citing websites as an information source, greater trust in healthcare providers (aOR: 1.65, 95% CI 1.26 – 2.15) and government communications (aOR: 0.69, 95% CI 0.55 – 0.86), and higher perceived risk of COVID-19 infection (aOR: 1.25, 95% CI 1.10 – 1.42). Factors associated with *acceptance* to take Paxlovid include male gender, citing trust in healthcare providers (aOR: 1.49, 95% CI 1.11 – 1.99) and government communications (aOR: 1.38, 95% CI 1.09 – 1.76), and higher perceived severity of COVID-19 (aOR: 1.23, 95% CI 1.07 – 1.42). Factors associated with *initiative* to request Paxlovid include male gender, having pre-existing diabetes and higher perceived severity of COVID-19 (aOR: 1.24, 95% CI 1.09 – 1.40). The most common reasons for why respondents might not take Paxlovid were concerns about side effects (64%), concerns about costs (29%), and the perception that COVID-19 is a mild (25%).

**Conclusion:**

The majority of our respondents would take Paxlovid if it was prescribed to them, but a much smaller proportion would have the initiative to request for this. Key factors that may influence uptake are COVID-19 threat perceptions, trust in healthcare and government, and perceptions of the drug’s side effects and cost.

**Supplementary Information:**

The online version contains supplementary material available at 10.1186/s12889-024-19687-0.

## Introduction

The COVID-19 pandemic prompted the development of numerous tools to counter the newly emerged severe acute respiratory syndrome coronavirus 2 (SARS-CoV-2). Amongst these were oral antiviral drugs, including nirmatrelvir/ritonavir (Paxlovid). When administered early, Paxlovid can shorten recovery time, reduce the burden of long COVID-19, alleviate pressure on hospitals during epidemics, and save lives in high-risk groups and those with medical risk factors [1]. In Singapore, Paxlovid is recommended for individuals aged 50 years [2] and older and those with underlying health conditions and until 27 March 2024 [3] was fully subsidised for eligible patients [4, 5]. Should a new COVID-19 variant with both greater severity and enhanced immune escape properties emerge, it will likely be challenging to develop and deploy updated COVID-19 vaccines in time to mitigate the potential impact [6–8]. In such a situation, Paxlovid may be a critical intervention not just for the groups currently considered vulnerable but also the wider population.

However, despite its accessibility [9], the use of Paxlovid in Singapore remains reportedly low, even in vulnerable groups [5]. A study conducted by Wee et al. [10] in 2022 showed that only 2.7% of notified cases aged 60 and above received Paxlovid. Underutilisation and missed opportunities for early treatment may arise due to patients’ hesitancy to take COVID-19 antiviral medication as advised [11]. Several articles have postulated that factors contributing to hesitancy towards a recommended intervention like antivirals includes lack of prior awareness, reduced perceptions of COVID-19 threat and severity, inadequate trust in the healthcare system or messaging about the drug, and perceptions about Paxlovid's efficacy, side effects and cost [9, 11–16]. In addition, medical professionals may also be reluctant to prescribe Paxlovid [17], in which case it is also worthwhile to ascertain if these factors are important drivers of a patient’s initiative to request Paxlovid from their doctors. However, there has been little empirical data examining how these factors specifically affect utilization of Paxlovid, and none in the context of COVID-19 in Singapore.

To better understand acceptance and use of Paxlovid as a COVID-19 antiviral treatment in Singapore, we conducted a survey using an ongoing community-based cohort (SOCRATES). We aimed to identify factors associated with participants’ awareness of Paxlovid, their acceptance of Paxlovid should it be prescribed, and their initiative to request Paxlovid if it is not offered by their healthcare provider. We also document possible reasons as to why our participants would not take Paxlovid.

## Methods

### Study design

The SOCRATES (Strengthening Our Community's Resilience Against Threats from Emerging Infections) research study was initiated before the COVID-19 pandemic in February 2019, specifically to assess public perceptions and response to the threat from and our interventions against infectious diseases. The intent was to set up a pre-enrolled cohort recruited from the public that could be efficiently surveyed on issues pertinent to circulating infectious diseases and allow rapid and repeated surveys during a public health emergency caused by an emerging infection, as was the case with COVID-19.

The design and setting up of the SOCRATES study have previously been described [18]. Briefly, we recruited participants aged 16 years and above into the SOCRATES cohort through various methods, including invitations to participants of other research studies, door-to-door recruitment, and word-of-mouth referrals. Participants could complete the surveys in English or key local languages (Malay and Mandarin).

Enrolment was performed through interviews conducted in person or via video-conferencing. Sociodemographic characteristics and pre-existing chronic illness were also collected at the enrolment interview. Subsequent follow-up surveys were conducted via FormSG (https://www.form.gov.sg), an online platform managed by the Singapore Government. The use of online methods for interviews and follow-up surveys allowed us to continue study-related activities in the face of restrictions imposed to minimise the spread of COVID-19.

### Survey on Paxlovid and factors influencing awareness and uptake

Following the government’s announcement on 31^st^ January 2022 [5] on the introduction of Paxlovid to Singapore through primary care providers, and that it would be fully subsidised for adults aged 60 years and older as well as those with selected medical conditions (those at risk of developing severe disease, have active cancer or serious heart, lung or kidney disorders, or are on ongoing immunosuppressive treatment) [5], we collected our data about Paxlovid between 29^th^ August and 6th September 2022 as part of the 36^th^ survey wave launched in our cohort.

To assess *awareness* of Paxlovid, *acceptance* towards taking Paxlovid and the *initiative* to request Paxlovid, a single questionnaire item was used for each. These three items were prefaced by a short preamble text (Table S1) taken from an online news article about Paxlovid rollout in Singapore [19]. Responses were regrouped where needed into binary outcomes as detailed in Table S1.

To investigate factors potentially associated with uptake, we used data collected on enrolment into our cohort for respondent characteristics such as current age, ethnicity, gender, highest education level, employment status, household income, and presence of common chronic illnesses (specifically diabetes and hypertension). We also used our participant’s previous survey responses (about 15 months before the survey on Paxlovid) to investigate the role of trust in the healthcare system (*“Our healthcare institutions, doctors, nurses and other healthcare professionals will be able to provide appropriate medical treatment to you if you contract the COVID-19 infection during the outbreak”*) and trust in government communications ("*The authorities will adequately communicate facts and information about COVID-19 to the public*"), with responses captured on a 4-point Likert scale, ranging from "Strongly agree" to "Strongly disagree". Additional questionnaire items for probing acceptance of Paxlovid were based on factors postulated by others [9, 11–16] and the conceptual framework of the Health Belief Model [20]. Participants were asked about their perceived susceptibility to being infected ("*I believe there is a strong likelihood I will contract COVID-19″*) and severe COVID-19 if infected ("*I believe that if I were to contract COVID-19 it would have serious consequences to my health"*) on a 5-point Likert scale ("Strongly agree" to "Strongly disagree”). In addition, respondents were asked to make multiple selections from a list of reasons why they may not take Paxlovid, including items representing perceived barriers (costs, side effects, inconvenience) and benefits (or lack thereof, such as perceived ineffectiveness of Paxlovid).

### Data analysis

We explored in three separate analyses if demographic characteristics and attitudes and perceptions were associated with the three previously defined outcome variables representing *awareness*, *acceptance* and *initiative*. Univariate and multivariable logistic regression was used to identify factors associated with the above binary outcomes, with results presented as crude and adjusted odds ratios (ORs), and *p*-values of < 0.05 considered statistically significant. In the regression analyses, demographic factors were coded as categorical variables. Questions regarding perceptions of COVID-19 and trust were modelled as scores. We had previously performed some validation for the questions used for COVID-19 perceptions [21], and examination of the validity of our questions on trust is described in our supplementary material (see Table S2). The multivariable models included all variables from the univariate analyses. To assess if this resulted in multicollinearity, we ran Ordinary Least Squares regression (OLS) regression with the same predictors in the models, using a Variance Inflation Factor (VIF) threshold of > 10 to indicate if there was severe collinearity between any of the predictors [22]. Goodness of fit for the multivariable models was assessed using the Hosmer–Lemeshow with 10 groups [23, 24].

All statistical analysis was performed on STATA 15 for Windows.

## Results

### Demographics of the Paxlovid survey respondents

Among the 2136 total participants in the SOCRATES cohort, there were 1432 respondents (60% female, mean age = 47.9 years, response rate = 68%) for the survey on Paxlovid (Table 1), of which 137 (10%) were from door-to-door recruitment, 580 (41%) from other cohorts, and 715 (50%) from referrals. The majority of respondents identified as Chinese (89%), held tertiary education (59%), and resided in 4–5 bedroom publicly owned flats (57%). About a third had a monthly household income of less than $5,000 (33%). Those who responded to the Paxlovid survey were reasonably similar to the rest of the cohort. However, minority ethnic groups were under-represented relative to population data from the Singapore Department of Statistics, as were individuals with fewer years of education (14% with ‘O’ / ‘N’ level and below amongst respondents versus 47% in national data). Most of the respondents either strongly agreed or agreed that the healthcare system and communications by the authorities regarding COVID-19 could be trusted (97% and 89% respectively, see Table S3). Levels of trust decreased slightly over serial surveys, and we used the respondent’s most recent response to this question in our multivariable analyses. In survey responses collected in August 2022, more than half (54%) either strongly agreed or agreed that they were likely to contract COVID-19, with 40% also believing contracting COVID-19 would have serious consequences on their health (Table S4).

**Table 1:** Demographics of participants (SOCRATES cohort vs Population data 2022)

Demographics	SOCRATES cohort		Respondents to survey on paxlovid		Population data (Yr 2022)^a^
	**Number of participants**	**%**	**Number of participants**	**%**	**%**
Age, years (as at 31 Dec 2022)					
Below 30	376	18%	201	14%	20%
30—39	455	21%	277	19%	18%
40—49	403	19%	271	19%	18%
50—59	396	19%	300	21%	17%
60 and above	506	24%	383	27%	28%
Gender					
Male	863	40%	571	40%	49%
Female	1273	60%	861	60%	51%
Ethnicity					
Chinese	1859	87%	1281	89%	74%
Malay	72	3%	31	2%	14%
Indian	151	7%	85	6%	9%
Others	54	3%	35	2%	3%
Education^b^					
'O' / 'N' level & below	313	15%	198	14%	47%
'A' level / Polytechnic diploma	623	29%	387	27%	17%
University / Post-graduate	1201	56%	847	59%	36%
Housing type					
Publicly owned flat with ≤ 3 rooms	306	14%	210	15%	24%
Publicly owned flat with 4–5 rooms	1250	59%	811	57%	54%
Privately owned property	580	27%	411	29%	22%
Monthly household income (SGD)^c^					
≤ 4999	686	32%	475	33%	33%
5000–8999	578	27%	376	26%	19%
≥ 9000	870	41%	579	40%	48%
Preexisting conditions					
Diabetes	98	5%	66	5%	-
Hypertension	214	10%	151	11%	-
No Diabetes nor Hypertension	1875	88%	1251	87%	

^a^Taken from singstat.gov.sg (Figures may not add up to the totals due to rounding)

^b^Population education data restricted to 25yo and older because that what is available

^e^Socrates cohort data includes 2 individuals who did not disclose household income

**Fig.1:**
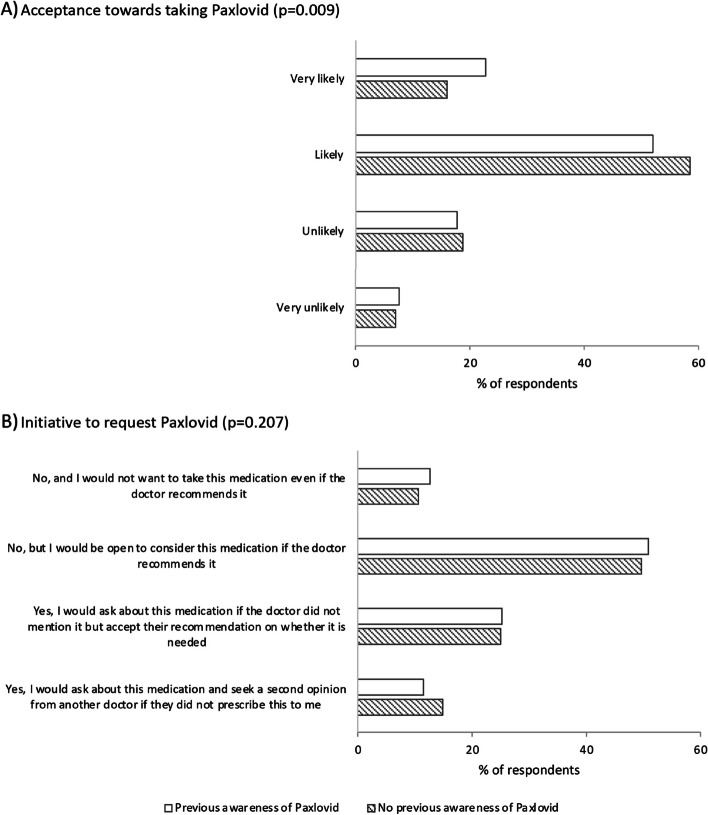
*Acceptance* towards taking Paxlovid if prescribed (**A**), and *Initiative* to request Paxlovid (**B**), stratified by whether the respondents’ previous *awareness* of Paxlovid. Pearson chi-square test was used to generate the *p*-values in the figure

More than half (54%) of respondents had heard about Paxlovid before our survey. Figure 1A indicates significant but subtle differences in acceptance towards Paxlovid by prior awareness (*p* = 0.009). Those with prior awareness had a higher proportion who were “very likely” to take Paxlovid if prescribed compared to those who had not previously heard of Paxlovid (23% vs 16%). However, when considering those who were “very likely” and “likely” to take Paxlovid as those with higher acceptance, then proportions were similar regardless of whether they were previously aware (75%) or not (74%). Overall, 38% would take the initiative to request Paxlovid if not suggested by their doctor, either by asking their doctor (25%) or even seeking a second opinion from another healthcare provider (13%). Figure 1B indicates that these proportions did not vary significantly by prior awareness of Paxlovid. However, if we assume that prior awareness of Paxlovid is needed for a patient to take the initiative to request Paxlovid from a doctor who did not offer it, then only 19.9% of all respondents would do so.

We next performed a series of binary logistic regression models to explore factors associated *awareness* of, *acceptance* towards taking, and the *initiative* to request Paxlovid.

All three multivariable models had reasonably good fit, with Hosmer–Lemeshow χ2 [10] values of 8.77 (*p* = 0.5539), 4.90 (*p* = 0.8979), and 8.77 (*p* = 0.5539) respectively. Also, amongst the predictors included, the highest VIF was 4.04, indicating an absence of multicollinearity. The multivariable regression model for awareness (Table 2) showed significant association for age, education, trust in healthcare providers, trust in government communications and likely to get COVID-19 infection. As compared to respondents aged below 30, significant association for prior awareness were observed for respondents aged 40–49 (aOR: 2.03, 95% CI 1.28 – 3.23), 50–59 years old (aOR: 2.47, 95% CI 1.55 – 3.94) and 60 and above (aOR: 3.62, 95% CI 2.23 – 5.88), indicating successively higher Paxlovid awareness among older age groups. Those with more years of education also had significantly greater awareness of Paxlovid, as did respondents who predominantly relied on websites as their preferred information source (aOR: 1.65, 95% CI 1.27 – 2.14) relative to those who did not rely on websites. Those who strongly agreed that healthcare providers could be trusted were more likely to have prior awareness of Paxlovid. When modelled as a score, the level of trust in healthcare providers was significantly associated with having prior awareness (OR: 1.65, 95% CI 1.26 – 2.15). Conversely, increasing trust in government communications was associated with lower proportions with prior awareness (aOR: 0.69, 95% CI 0.55 – 0.86). Those who perceived a higher likelihood of COVID-19 infection were significantly more likely to have prior awareness of Paxlovid (aOR: 1.25, 95% CI 1.10 – 1.42).

**Table 2:** Multivariable regression analysis showing factors associated with having *awareness of Paxlovid*

**Category**		**% of respondents**^**a**^	** Crude Odds Ratio (CI)**^**b**^	***p*-value**	**Adjusted Odds Ratio (CI)**^**b**^	***p*-value**
Recruitment method	Door to door	56%	reference		reference	
	Referral	49%	0.77 (0.5, 1.18)	0.229	0.89 (0.55, 1.43)	0.635
	Other cohort	59%	1.11 (0.72, 1.7)	0.635	0.9 (0.56, 1.46)	0.679
Age group	Below 30	37%	reference		reference	
	30 - 39	49%	1.63 (1.09, 2.46)	0.018	1.52 (0.97, 2.37)	0.066
	40 - 49	55%	2.07 (1.38, 3.09)	<0.001	2.03 (1.28, 3.23)	0.003
	50 - 59	58%	2.28 (1.53, 3.39)	<0.001	2.47 (1.55, 3.94)	<0.001
	60 and above	62%	2.75 (1.87, 4.03)	<0.001	3.62 (2.23, 5.88)	<0.001
Gender	Male	54%	reference		reference	
	Female	54%	1.04 (0.83, 1.3)	0.759	1.22 (0.95, 1.57)	0.116
Race	Chinese	55%	reference		reference	
	Malay	33%	0.4 (0.18, 0.91)	0.028	0.64 (0.27, 1.53)	0.316
	Indian	46%	0.68 (0.43, 1.07)	0.095	0.79 (0.48, 1.29)	0.343
	Others	52%	0.86 (0.42, 1.76)	0.684	0.79 (0.37, 1.68)	0.534
Highest education	'O' / 'N' level & below	42%	reference		reference	
	'A' level / Polytechnic diploma	51%	1.44 (1, 2.08)	0.051	1.74 (1.16, 2.61)	0.007
	University / Post-graduate	58%	1.93 (1.38, 2.69)	<0.001	2.22 (1.51, 3.28)	<0.001
Housing type	Privately owned property	54%	reference		reference	
	Publicly owned flat with ≤ 3 rooms	54%	0.98 (0.68, 1.4)	0.9	1.33 (0.88, 2.01)	0.180
	Publicly owned flat with 4–5 rooms	55%	0.94 (0.73, 1.22)	0.642	1.26 (0.94, 1.68)	0.121
Monthly household income (SGD)^c^	≤ 4999	52%	reference		reference	
	5000–8999	54%	1.08 (0.81, 1.44)	0.601	1.09 (0.8, 1.5)	0.589
	≥ 9000	56%	1.15 (0.88, 1.49)	0.301	1.1 (0.81, 1.5)	0.536
*Preexisting conditions:*						
Diabetes	No	32%	reference		reference	
	Yes	68%	1.89 (1.07, 3.34)	0.029	1.74 (0.92, 3.26)	0.087
Hypertension	No	43%	reference		reference	
	Yes	57%	1.15 (0.79, 1.68)	0.459	0.85 (0.55, 1.31)	0.459
*Where do you usually get information about outbreaks of infectious diseases in Singapore?*^d^						
TV		56%	1.19 (0.94, 1.5)	0.147	1.01 (0.77, 1.32)	0.943
Radio		57%	1.2 (0.94, 1.55)	0.147	1.02 (0.77, 1.36)	0.874
Print media (e.g. newspapers / fliers / notice boards / posters / banners / bus-stop boards)		59%	1.43 (1.14, 1.79)	0.002	1.22 (0.95, 1.57)	0.126
Family / relatives		55%	1.07 (0.85, 1.33)	0.576	1.14 (0.84, 1.56)	0.405
Friends / colleagues		54%	1.01 (0.8, 1.26)	0.946	0.9 (0.66, 1.23)	0.512
Social media (e.g. Facebook / Instagram / Twitter / Straits Times / Facebook Page)		53%	0.75 (0.57, 0.98)	0.035	0.79 (0.59, 1.07)	0.126
Websites (e.g. Google / Yahoo / MSN / Straits Times Online / MOH)		58%	1.66 (1.3, 2.11)	<0.001	1.65 (1.27, 2.14)	<0.001
Trust in healthcare providers^e,f^	Strongly disagree	57%	1.32 (1.08, 1.62)	0.007	1.65 (1.26, 2.15)	<0.001
	Disagree	48%				
	Agree	51%				
	Strongly agree	59%				
Trust in government communications^e,f^	Strongly disagree	58%	0.91 (0.77, 1.08)	0.274	0.69 (0.55, 0.86)	0.001
	Disagree	64%				
	Agree	53%				
	Strongly agree	54%				
Likely to get COVID-19 infection^f^	Strongly disagree	42%	1.14 (1.02, 1.28)	0.019	1.25 (1.1, 1.42)	0.001
	Somewhat disagree	50%				
	Neither agree nor disagree	51%				
	Somewhat agree	58%				
	Strongly agree	56%				
Serious consequences if infected^f^	Strongly disagree	66%	0.94 (0.85, 1.05)	0.274	0.9 (0.8, 1.02)	0.108
	Somewhat disagree	57%				
	Neither agree nor disagree	51%				
	Somewhat agree	53%				
	Strongly agree	56%				

^a^Percentages shown are row percentages, and are based on all respondents to the 39^th^ survey wave on Paxlovid who had valid responses to that variable, and includes 1432 observations unless otherwise mentioned

^b^Crude Odds Ratios and Adjusted Odds Ratios include only 1240 observations (excludes 2 respondents with missing household income data and another 190 respondents who did not have previous survey responses on trust in government and healthcare providers), with Adjusted Odds Ratios based on multivariable analyses adjusting for all variables in the above table

^c^Excludes 2 respondents who did not disclose household income

^d^Variables are in binary format where the reference category is those who did not cite that information source

^e^Excludes 190 respondents who did not have previous survey responses on trust in government and healthcare providers

^f^These variables are analysed on an ordinal scale format

Table 3 gives the results from our multivariable regression model to predict respondents’ acceptance of Paxlovid. The model showed significant association for gender, trust in healthcare providers, trust in government communications and serious consequences if infected. Female gender was significantly associated with lower acceptance (aOR: 0.42, 95% CI 0.31 – 0.57) when compared to males, but no significant associations were found for other socio-demographic factors. Higher levels of trust in healthcare providers (aOR: 1.49, 95% CI 1.11 – 1.99) and government communications (aOR: 1.38, 95% CI 1.09 – 1.76) were significantly associated with higher acceptance. Those who perceived more serious consequences from COVID-19 (aOR: 1.23, 95% CI 1.07 – 1.42) also had higher acceptance.

**Table 3:** Multivariable regression analysis showing factors associated with the *acceptance towards taking Paxlovid*

**Category**		**% of respondents**^**a**^	**Crude Odds Ratio (CI)**^**b**^	***p*-value**	**Adjusted Odds Ratio (CI)**^**2**^	***p*-value**
Recruitment method	Door to door	75%	reference		reference	
	Referral	73%	0.89 (0.54, 1.44)	0.627	1.12 (0.66, 1.9)	0.685
	Other cohort	75%	1 (0.61, 1.63)	0.985	1.32 (0.77, 2.27)	0.309
Age group	Below 30	77%	reference		reference	
	30—39	74%	0.88 (0.56, 1.41)	0.606	0.86 (0.52, 1.43)	0.562
	40—49	74%	0.87 (0.55, 1.37)	0.545	0.76 (0.45, 1.28)	0.300
	50—59	75%	0.91 (0.58, 1.43)	0.675	0.78 (0.46, 1.32)	0.347
	60 and above	72%	0.78 (0.5, 1.19)	0.248	0.65 (0.38, 1.12)	0.123
Gender	Male	83%	reference		reference	
	Female	68%	0.43 (0.32, 0.56)	< 0.001	0.42 (0.31, 0.57)	< 0.001
Race	Chinese	74%	reference		reference	
	Malay	85%	2.07 (0.71, 6.04)	0.183	2.17 (0.71, 6.64)	0.174
	Indian	72%	0.93 (0.56, 1.55)	0.789	0.76 (0.44, 1.31)	0.326
	Others	81%	1.5 (0.61, 3.69)	0.378	1.46 (0.57, 3.71)	0.429
Highest education	'O' / 'N' level & below	71%	reference		reference	
	'A' level / Polytechnic diploma	72%	1.04 (0.7, 1.56)	0.84	0.93 (0.6, 1.45)	0.761
	University / Post-graduate	75%	1.22 (0.85, 1.76)	0.282	1 (0.65, 1.53)	0.988
Housing type	Privately owned property	76%	reference		reference	
	Publicly owned flat with ≤ 3 rooms	73%	1.06 (0.7, 1.61)	0.79	1.23 (0.76, 1.99)	0.389
	Publicly owned flat with 4–5 rooms	75%	0.91 (0.68, 1.22)	0.525	0.88 (0.64, 1.22)	0.452
Monthly household income (SGD)^c^	≤ 4999	72%	reference		reference	
	5000–8999	75%	1.19 (0.86, 1.65)	0.303	1.14 (0.8, 1.63)	0.461
	≥ 9000	75%	1.14 (0.85, 1.53)	0.385	1.05 (0.75, 1.47)	0.783
*Preexisting conditions:*						
Diabetes	No	25%	reference		reference	
	Yes	75%	1.09 (0.59, 2.02)	0.783	1.06 (0.53, 2.12)	0.863
Hypertension	No	23%	reference		reference	
	Yes	77%	1.18 (0.76, 1.82)	0.464	0.96 (0.58, 1.59)	0.883
*Where do you usually get information about outbreaks of infectious diseases in Singapore?*^d^						
TV		74%	1.01 (0.77, 1.31)	0.945	1.03 (0.76, 1.4)	0.853
Radio		72%	0.89 (0.68, 1.18)	0.437	0.91 (0.66, 1.25)	0.564
Print media (e.g. newspapers / fliers / notice boards / posters / banners / bus-stop boards)		76%	1.23 (0.96, 1.59)	0.107	1.31 (0.98, 1.75)	0.065
Family / relatives		71%	0.78 (0.6, 1)	0.049	0.77 (0.55, 1.09)	0.147
Friends / colleagues		73%	0.9 (0.69, 1.16)	0.41	1.12 (0.79, 1.58)	0.535
Social media (e.g. Facebook / Instagram / Twitter / Straits Times / Facebook Page)		74%	0.95 (0.7, 1.29)	0.738	0.89 (0.64, 1.25)	0.507
Websites (e.g. Google / Yahoo / MSN / Straits Times Online / MOH)		74%	0.98 (0.74, 1.28)	0.862	0.93 (0.69, 1.25)	0.622
Trust in healthcare providers^e,f^	Strongly disagree	29%	1.79 (1.43, 2.26)	< 0.001	1.49 (1.11, 1.99)	0.008
	Disagree	48%				
	Agree	71%				
	Strongly agree	80%				
Trust in government communications^e,f^	Strongly disagree	45%	1.54 (1.28, 1.86)	< 0.001	1.38 (1.09, 1.76)	0.008
	Disagree	64%				
	Agree	74%				
	Strongly agree	79%				
Likely to get COVID-19 infection^f^	Strongly disagree	47%	1.26 (1.11, 1.43)	< 0.001	1.15 (0.99, 1.33)	0.059
	Somewhat disagree	73%				
	Neither agree nor disagree	72%				
	Somewhat agree	76%				
	Strongly agree	79%				
Serious consequences if infected^f^	Strongly disagree	52%	1.21 (1.07, 1.36)	0.002	1.23 (1.07, 1.42)	0.004
	Somewhat disagree	75%				
	Neither agree nor disagree	73%				
	Somewhat agree	75%				
	Strongly agree	81%				
Have you heard about the use of Paxlovid medication to reduce COVID-19 severity?		54%	1.01 (0.78, 1.3)	0.966	1 (0.76, 1.32)	0.992

^a^Percentages shown are row percentages, and are based on all respondents to the 39th survey wave on Paxlovid who had valid responses to that variable, and includes 1432 observations unless otherwise mentioned

^b^Crude Odds Ratios and Adjusted Odds Ratios include only 1240 observations (excludes 2 respondents with missing household income data and another 190 respondents who did not have previous survey responses on trust in government and healthcare providers), with Adjusted Odds Ratios based on multivariable analyses adjusting for all variables in the above table

^c^ Excludes 2 respondents who did not disclose household income

^d^Variables are in binary format where the reference category is those who did not cite that information source

^e^Excludes 190 respondents who did not have previous survey responses on trust in government and healthcare providers

^f^ These variables are analysed on an ordinal scale format

Regarding the initiative to request Paxlovid, Table S5 shows that females were less likely (aOR: 0.50, 95% CI 0.39 – 0.64), while those who perceived more serious consequences from COVID-19 (aOR: 1.24, 95% CI 1.09 – 1.40) were more likely to take the initiative to request Paxlovid.

**Fig. 2:**
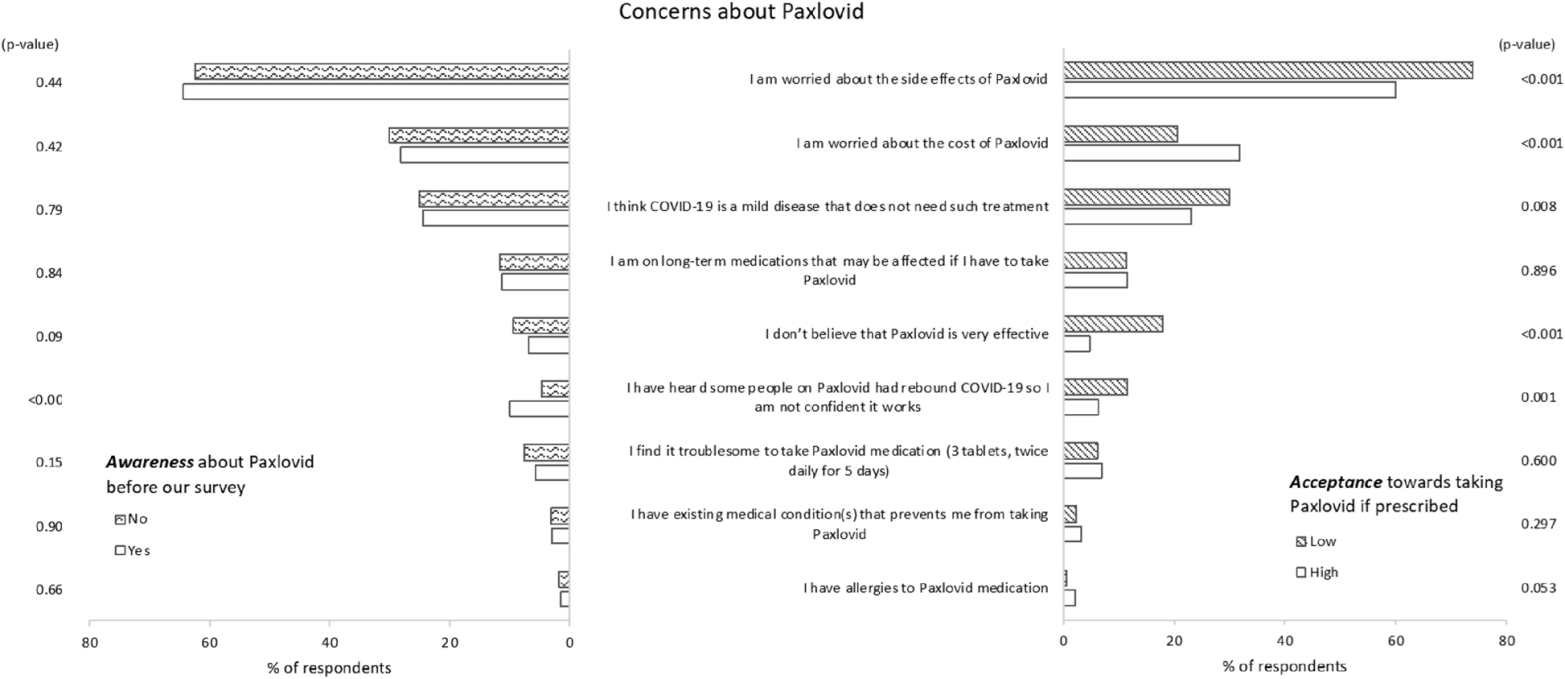
Concerns about taking Paxlovid

Figure 2 ranks several concerns from our respondents about taking Paxlovid. The top three were potential side effects (64%), the cost of Paxlovid (29%), and perceptions that COVID-19 is not severe (25%). The proportions for all three differed significantly by acceptance but not prior awareness. In those with higher acceptance of Paxlovid, cost was more frequently (32% vs 21%, *p* < 0.001), while side effects (60% vs 74%, *p* < 0.001) and perceptions that COVID-19 was a mild disease (23% vs 30%, *p* = 0.008) were less frequently reported than in those with lower acceptance. Amongst those with lower acceptance towards Paxlovid, a substantial proportion believed that Paxlovid was not effective (18% vs 5% in those with higher acceptance, *p* < 0.001).

Supplementary table S6 shows that more women than men were concerned about side effects (aOR: 1.37, 95% CI 1.07 – 1.76), while those with higher trust in healthcare providers (aOR: 0.78,, 95% CI 0.60 – 1.02) were less likely to be so. In table S7, individuals aged 60 and above were less likely to be concerned about costs (aOR: 0.55, 95% CI 0.33 – 0.91). Respondents with lower trust in government communications were more likely to be concerned that Paxlovid is not effective (aOR: 0.46, 95% CI 0.33 – 0.64).

## Discussion

Our findings suggest that the delivery of Paxlovid in our setting is highly dependent on having primary care doctors diagnose COVID-19 and recommend it to their patients. Only a slim majority of respondents had previously heard of Paxlovid as an effective treatment for reducing COVID-19 severity, and less than 20% were both sufficiently informed and had the initiative to request it. On the other hand, about three-quarters would likely or very likely take Paxlovid if prescribed, and acceptance was not markedly different by prior awareness.

Older age groups and more years of education were associated with greater awareness; the former were the target group for messaging on the benefits of treatment, and the latter may have greater exposure and better understanding and recall of information about Paxlovid. During the 2009 H1N1 influenza pandemic, Fietjé et al. found that patients with higher education levels were more likely to receive off-guideline prescriptions of oseltamivir [25]. Greater awareness about antiviral agents in more highly educated patients may have been a contributing factor, given the correlation we observed between better education and awareness of Paxlovid.

As for acceptance towards Paxlovid, risk perceptions regarding COVID-19 and respondents’ levels of trust towards government messaging and healthcare providers were important and potentially modifiable factors. We also found significant differences in the concerns about taking Paxlovid between respondents with higher and lower acceptance.

Although a substantial majority said they would accept Paxlovid if it was prescribed to them, it is still relevant to highlight factors that prime a population for greater uptake. The relationship between some of these factors and concerns about taking Paxlovid are possibly applicable to settings beyond Singapore. Of the demographic factors, the only significant association was that females had lower acceptance. Interestingly, women were also more likely to express concerns about unwanted side effects (Refer to supplementary Table S6). In general, women have been found to experience more medication side effects than men [26–28], and their being aware of this, possibly through experiences with other medications, may have caused them to adopt a more cautious view of new medications, including COVID-19 antivirals. Moreover, there are genuine concerns that molnupiravir, the other COVID-19 antiviral available in Singapore, can cause DNA mutations and hence birth defects in a developing foetus [29], and this could have led to wider concerns amongst women about COVID-19 antiviral use in general.

The other notable factors significantly associated with acceptance arose from respondents’ perceptions. Firstly, as with our previous work [18], greater trust in government communications was associated with higher acceptance and being less likely to cite the effectiveness of Paxlovid as a concern. Moreover, although trust in government communications was strongly correlated with trust in their healthcare providers (Spearman’s *r* = 0.5735, *p* < 0.001, for the two scores), the latter was also independently associated with higher acceptance. Those with greater trust in healthcare providers were also less likely to be concerned about side effects. The public often relies on guidance from government agencies and healthcare providers when making informed decisions about their health. Trust in government communications may reflect confidence in the overall public health response and the regulatory processes ensuring the safety and efficacy of medications such as Paxlovid [30, 31]. Complementary to this, healthcare providers, acting as the primary point of contact for medical care, are positioned to recommend appropriate treatments based on individual patient needs while ensuring that prescribed medications are reasonably safe given each patient’s medical history.

Kritzinger et al. [32] suggested that perceptions of the government's appropriateness in handling the crisis play an essential role in people's trust, and a consistent approach is needed to establish trust with the public over time. On the other hand, Amara et al. [33] discovered that respondents would already generally trust their healthcare providers, and we found that trust in healthcare providers was positively associated with awareness of Paxlovid. However, as healthcare encounters typically follow the advent of an illness episode, there is a potential limitation in relying on healthcare providers to inform their patients about Paxlovid. An earlier survey in our cohort (unpublished data) found that knowing about effective treatments can predispose individuals to seek medical care should they develop COVID-like symptoms. Therefore, while this survey found that prior awareness did not have a substantial effect on acceptance, awareness may still have an indirect effect on uptake by incentivising symptomatic individuals to seek care, particularly since Paxlovid should ideally be given early in the course of illness. However, those with higher trust in government communications had lower proportions with prior awareness. While somewhat unexpected, we speculate that that Paxlovid was not sufficiently promoted in government messaging platforms, so that those who were more dependent on such messaging may thus have been less aware. To fully leverage on the public’s trust in healthcare providers and government communications, it would be necessary to firstly communicate messages that raise awareness so as to drive access to healthcare, then follow through by having healthcare providers recommend treatment to a receptive population.

Not unexpectedly, respondents who perceived that contracting COVID-19 could have severe consequences were more likely to have higher acceptance towards Paxlovid as well as have the initiative to request Paxlovid. Moreover, the perception that COVID-19 is a mild disease not needing such treatment was the third most frequent concern about Paxlovid, and significantly more common amongst those with lower acceptance towards Paxlovid. Perceptions of COVID-19 severity are not static. Over serial surveys, we observed a decrease in the proportion of people who strongly agreed or agreed that COVID-19 infection would have severe consequences (see Table S4). This may cause acceptance towards interventions like Paxlovid to decrease over time. Changing perceptions of COVID-19 severity are not unjustified since the omicron subvariants which have been dominant since early 2022, have been shown to be less severe than the preceding delta COVID-19 variant [34, 35]. Also, after Singapore achieved high levels of vaccine coverage, there was an intentional pivot in messaging away from preventing infection to an emphasis on “living with COVID-19” [36–38]. Such messaging could also have contributed to the shifts in perceptions. However, new COVID-19 variants continue to emerge, exhibiting varying degrees of resistance to immunity conferred by past infection and existing COVID-19 vaccines. One modelling study exploring vaccine allocation for facilitating an effective response to COVID-19 also highlighted how antiviral treatments might be necessary to supplement vaccination efforts, mainly when vaccine supply is limited or for COVID-19 variants that are partially resistant to current vaccines [39]. Moreover, transmission models of SARS-CoV-2 also suggest that antiviral treatments may mitigate transmission of COVID-19 [40]. Should a COVID-19 variant emerge with high levels of immune escape and more significant morbidity and mortality, antiviral treatments may need to be deployed more widely beyond older age groups and medically vulnerable individuals. If so, this would require an appropriately timed change of messaging about which groups should take Paxlovid, alongside an updated emphasis on imminent risks of infection and greater likelihood of severe disease to drive awareness of and acceptance towards antiviral use, respectively.

While there is a paucity of literature specific to acceptance of oral COVID-19 antivirals, Mercadante and Law [41] found that concerns about side effects, inconvenience, and lack of confidence in the authorities are possibly some of the perceived barriers for the uptake of influenza and COVID-19 vaccines. In addition, perceived susceptibility to infection and perceived severity of the disease also significantly influenced vaccination intent. These factors are somewhat similar to what we found for antiviral medications. Addressing these perceptions may hence benefit both uptake of relevant vaccines as oral antiviral agents.

Some limitations apply when generalising our findings to the broader population. Firstly, it must be acknowledged that compared to the Singapore population, ethnic minorities, individuals with fewer years of education, and those living in smaller publicly owned residences are under-represented amongst our respondents. Some of these are underserved populations with poorer health literacy who also face more significant challenges in accessing health services and antiviral medications. Our study may hence be over-estimating awareness and acceptance of antiviral use for COVID-19. Secondly, by the time of our survey in the second half of 2022, a substantial proportion of our population (estimated in another survey of the cohort to be about 61%) might already have seen a doctor for COVID-19. If so, they may have learned of Paxlovid through their healthcare providers, and awareness of Paxlovid prior to diagnosis may be lower than we estimated. Moreover, this could also have contributed to the observed association that those with greater trust in healthcare providers were more likely to be aware of Paxlovid. Another limitation of our study is the reliance on single-item self-reported data to measure *awareness* of, *acceptance* towards taking and the *initiative* to request Paxlovid. Self-reported data are susceptible to various forms of response bias, including social desirability and recall biases. These biases can lead to overestimation or underestimation of the true awareness, acceptance, and willingness levels. Future research could consider using multi-item scales that can provide more reliable and valid measures of these constructs. Additionally, incorporating objective measures, such as electronic health records or observational data of antiviral prescriptions, could enhance the accuracy of the findings.

## Conclusions

In summary, our study suggests that the levels of awareness of Paxlovid and the initiative to request this treatment from their doctors can be improved. Since most patients would accept Paxlovid if their doctors recommend it, increased uptake would largely depend on having our doctors recommend this to more of their patients. It will also be helpful to address common concerns about side effects, cost and effectiveness, but underpinning all such communications may be the level of trust in official channels and healthcare providers, which would need to be built over time. In addition, it is unsurprising but noteworthy that perceived severity was strongly associated with acceptance towards Paxlovid. Perceptions are likely influenced by both the epidemiology of circulating variants and how the risks associated with COVID-19 are communicated and received. Therefore, when circumstances are rapidly evolving, trust can be particularly valuable for re-calibrating public perceptions of risk and benefit, so as to drive awareness of and acceptance towards antiviral agents and other interventions.

### Supplementary Information

Supplementary Material 1

## Data Availability

Please be advised that the information outlined in this article may be made available upon request from the designated corresponding author. However, we must emphasise that such disclosure is subject to the applicable legal and regulatory requirements, as well as at the sole discretion of the corresponding author.
