# Supplementary material for: Insights from a community-based survey on factors influencing acceptance and uptake of Paxlovid (nirmatrelvir and ritonavir) as a COVID-19 antiviral medication in Singapore
Source: BMC Public Health. 2024 Aug 28;24:2332. doi: 10.1186/s12889-024-19687-0 (PMC11351289; doi:10.1186/s12889-024-19687-0)
Supplement: Supplementary file 1 — Supplementary Material 1 [file 12889_2024_19687_MOESM1_ESM.docx]

Supplementary materials

Table S1 – Survey questions on Paxlovid (in wave 36):

| *On 31^st^ Jan 2022, Health Sciences Authority of Singapore approved Pfizer’s Paxlovid medication under Pandemic Special Access Route (PSAR) to treat people who are infected with COVID-19 and showed mild or moderate symptoms. Paxlovid medication is shown to reduce the risk of COVID-19 related hospitalisation or death by 88%. The medication consists of three tablets that have to be consumed twice daily for 5 consecutive days from onset of symptoms.*   1. Have you heard about the use of Paxlovid medication to reduce COVID-19 severity? *(****awareness*** *of Paxlovid)^1^*    - Yes    - No 2. Does knowing that there is a highly effective medication like Paxlovid, that can reduce COVID-19 severity, influence your decision to see a doctor when you have COVID-19 symptoms?    - I would see a doctor regardless of whether or not there is a medication that reduces COVID-19 severity    - Knowing that there is such a treatment makes me more likely to see a doctor when I develop symptoms    - Knowing that there is such a treatment makes me more likely to see a doctor, but only if I feel my symptoms are severe    - I would not be likely to see a doctor regardless of whether or not there is a medication that reduces COVID-19 severity 3. If you were infected with COVID-19, would you ask the healthcare provider whether you can be treated with Paxlovid medication? *(****initiative*** *to request Paxlovid)^2^*    - Yes, I would ask about this medication and seek a second opinion from another doctor if they did not prescribe this to me    - Yes, I would ask about this medication if the doctor did not mention it but accept their recommendation on whether it is needed    - No, but I would be open to consider this medication if the doctor recommends it    - No, and I would not want to take this medication even if the doctor recommends it 4. If you were infected with COVID-19 and the healthcare provider prescribes the Paxlovid medication to you, how likely are you to take these drugs? *(****acceptance*** *towards taking Paxlovid)^3^*    - Very likely    - Likely    - Unlikely    - Very unlikely 5. What are some of the reasons why you are unlikely to accept a doctor’s recommendation to take Paxlovid? <select all that apply>  - I think COVID-19 is a mild disease that does not need such treatment - I am worried about the side effects of Paxlovid - I am on long-term medications that may be affected if I have to take Paxlovid - I am worried about the cost of Paxlovid - I don’t believe that Paxlovid is every effective - I have heard some people on Paxlovid had rebound COVID-19 so I am not confident it works - I have existing medical condition(s) that prevents me from taking Paxlovid - I find it troublesome to take Paxlovid medication (3 tablets, twice daily for 5 days) - I have allergies to Paxlovid medication - Others, please specify: _________________________   NOTE:  ^1”^Awareness” of Paxlovid binary variable was derived from the options “Yes” coded as *True* and “No” coded as *False*.  ^2”^Initiative” to request for Paxlovid binary variable was derived from grouping the options “Yes, I would ask about this medication and seek a second opinion from another doctor if they did not prescribe this to me” and “Yes, I would ask about this medication if the doctor did not mention it but accept their recommendation on whether it is needed” which was then coded as *True,* and grouping “No, but I would be open to consider this medication if the doctor recommends it” and “No, and I would not want to take this medication even if the doctor recommends it” which was then coded as *False*.  ^3^Acceptance to take Paxlovid binary variable derived from grouping the options “Very likely” and “Likely” which was then coded as *True,* and grouping “Very unlikely” and “Unlikely” which was then coded as *False*. |
| --- |

Table S2 – Trust questionnaire scale with Cronbach's alpha test results

| Text of question | Variable name | Item-rest correlation | Average interitem correlation | alpha |
| --- | --- | --- | --- | --- |
| Our healthcare institutions, doctors, nurses and other healthcare professionals will be able to provide appropriate medical treatment to you if you contract the COVID-19 infection during the outbreak. | Trust in healthcare providers | 0.5928 | 0.7511 | 0.9005 |
| The authorities will adequately communicate facts and information about COVID-19 to the public. | Trust in government communications | 0.8056 | 0.6020 | 0.8194 |
| The authorities can respond effectively and swiftly to protect the health of the public by controlling outbreaks of the COVID-19. | Trust in response | 0.7771 | 0.6208 | 0.8308 |
| You trust the authorities. | Trust in authority | 0.7918 | 0.6111 | 0.8250 |
| While overall Cronbach alpha was high, there is some suggestion “Trust in healthcare providers” represents a different construct, as evidenced by its lower item-rest correlation (0.5928 versus 0.7771 to 0.8056 for the other 3 items). This is to be expected from the wording of the question which specifically mentions healthcare providers rather than the term “the authorities” used by the other three items. We hence chose to use two variables as predictors in our model, one representing “Trust in healthcare providers”, and the other being “Trust in government communications”. | | | | |

Table S3 – Distribution of responses for questions on trust for each survey wave:

Table S4 – Respondents’ risk perception about COVID-19 across surveys:

Table S5 – Multivariable regression analysis showing factors associated with the *initiative to request Paxlovid*

| **Category** |  | **% of respondents^1^** | **Crude Odds Ratio (CI)^2^** | **p-value** | **Adjusted Odds Ratio (CI)^2^** | **p-value** |  |
| --- | --- | --- | --- | --- | --- | --- | --- |
| Recruitment method | Door to door | 36% | reference |  | reference |  |  |
|  | Referral | 37% | 1.05 (0.68, 1.64) | 0.819 | 1.09 (0.68, 1.76) | 0.711 |  |
|  | Other cohort | 39% | 1.12 (0.72, 1.74) | 0.621 | 1.16 (0.72, 1.87) | 0.551 |  |
|  |  |  |  |  |  |  |  |
| Age group | Below 30 | 39% | reference |  | reference |  |  |
|  | 30 - 39 | 38% | 0.99 (0.66, 1.5) | 0.98 | 1.04 (0.66, 1.63) | 0.866 |  |
|  | 40 - 49 | 34% | 0.82 (0.55, 1.24) | 0.349 | 0.83 (0.52, 1.33) | 0.437 |  |
|  | 50 - 59 | 38% | 0.97 (0.65, 1.45) | 0.894 | 0.98 (0.61, 1.56) | 0.923 |  |
|  | 60 and above | 39% | 1.04 (0.71, 1.52) | 0.855 | 1.11 (0.68, 1.81) | 0.669 |  |
|  |  |  |  |  |  |  |  |
| Gender | Male | 48% | reference |  | reference |  |  |
|  | Female | 31% | 0.5 (0.39, 0.63) | <0.001 | 0.5 (0.39, 0.64) | <0.001 |  |
|  |  |  |  |  |  |  |  |
| Race | Chinese | 37% | reference |  | reference |  |  |
|  | Malay | 41% | 1.18 (0.54, 2.56) | 0.683 | 1.09 (0.48, 2.49) | 0.832 |  |
|  | Indian | 47% | 1.51 (0.95, 2.38) | 0.08 | 1.4 (0.87, 2.27) | 0.170 |  |
|  | Others | 42% | 1.24 (0.6, 2.55) | 0.568 | 1.24 (0.58, 2.63) | 0.581 |  |
|  |  |  |  |  |  |  |  |
| Highest education | 'O' / 'N' level & below | 39% | reference |  | reference |  |  |
|  | 'A' level / Polytechnic diploma | 38% | 0.93 (0.64, 1.35) | 0.697 | 0.88 (0.59, 1.32) | 0.543 |  |
|  | University / Post-graduate | 37% | 0.92 (0.66, 1.29) | 0.64 | 0.8 (0.54, 1.18) | 0.261 |  |
|  |  |  |  |  |  |  |  |
| Housing type | Privately owned property | 40% | reference |  | reference |  |  |
|  | Publicly owned flat with ≤ 3 rooms | 36% | 1.04 (0.72, 1.51) | 0.82 | 1.29 (0.85, 1.96) | 0.232 |  |
|  | Publicly owned flat with 4–5 rooms | 39% | 0.87 (0.67, 1.14) | 0.319 | 0.9 (0.67, 1.2) | 0.463 |  |
|  |  |  |  |  |  |  |  |
| Monthly household income (SGD)^3^ | ≤ 4999 | 36% | reference |  | reference |  |  |
|  | 5000–8999 | 34% | 0.91 (0.67, 1.23) | 0.533 | 0.95 (0.68, 1.31) | 0.737 |  |
|  | ≥ 9000 | 42% | 1.27 (0.97, 1.66) | 0.079 | 1.35 (0.99, 1.83) | 0.058 |  |
|  |  |  |  |  |  |  |  |
| *Preexisting conditions:* |  |  |  |  |  |  |  |
| Diabetes | No | 49% | reference |  | reference |  |  |
|  | Yes | 51% | 1.76 (1.03, 2.99) | 0.038 | 1.62 (0.89, 2.92) | 0.112 |  |
|  |  |  |  |  |  |  |  |
| Hypertension | No | 57% | reference |  | reference |  |  |
|  | Yes | 43% | 1.26 (0.87, 1.84) | 0.227 | 0.87 (0.57, 1.34) | 0.533 |  |
|  |  |  |  |  |  |  |  |
| *Where do you usually get information about outbreaks of infectious diseases in Singapore? ^4^* | |  |  |  |  |  |  |
| TV |  | 37% | 0.87 (0.69, 1.11) | 0.262 | 0.88 (0.67, 1.16) | 0.368 |  |
| Radio |  | 36% | 0.91 (0.7, 1.18) | 0.475 | 0.91 (0.68, 1.22) | 0.524 |  |
| Print media (e.g. newspapers / fliers / notice boards / posters / banners / bus-stop boards) | | 39% | 1.08 (0.86, 1.36) | 0.5 | 1.14 (0.88, 1.47) | 0.315 |  |
| Family / relatives |  | 37% | 0.97 (0.77, 1.22) | 0.804 | 1.11 (0.81, 1.53) | 0.511 |  |
| Friends / colleagues |  | 36% | 0.88 (0.7, 1.11) | 0.27 | 0.85 (0.62, 1.16) | 0.296 |  |
| Social media (e.g. Facebook / Instagram / Twitter / Straits Times / Facebook Page) | | 38% | 1.12 (0.85, 1.48) | 0.426 | 1.15 (0.85, 1.55) | 0.379 |  |
| Websites (e.g. Google / Yahoo / MSN / Straits Times Online / MOH) |  | 38% | 1.09 (0.85, 1.4) | 0.483 | 1.13 (0.86, 1.47) | 0.383 |  |
|  |  |  |  |  |  |  |  |
| Trust in healthcare providers ^5,6^ | Strongly disagree | 57% | 1.16 (0.94, 1.43) | 0.158 | 1.05 (0.81, 1.36) | 0.727 |  |
|  | Disagree | 32% |  |  |  |  |  |
|  | Agree | 36% |  |  |  |  |  |
|  | Strongly agree | 41% |  |  |  |  |  |
|  |  |  |  |  |  |  |  |
| Trust in government communications ^5,6^ | Strongly disagree | 33% | 1.15 (0.97, 1.37) | 0.108 | 1.15 (0.92, 1.43) | 0.218 |  |
|  | Disagree | 33% |  |  |  |  |  |
|  | Agree | 37% |  |  |  |  |  |
|  | Strongly agree | 41% |  |  |  |  |  |
|  |  |  |  |  |  |  |  |
| Likely to get COVID-19 infection ^6^ | Strongly disagree | 23% | 1.16 (1.03, 1.3) | 0.012 | 1.07 (0.94, 1.22) | 0.333 |  |
|  | Somewhat disagree | 39% |  |  |  |  |  |
|  | Neither agree nor disagree | 33% |  |  |  |  |  |
|  | Somewhat agree | 42% |  |  |  |  |  |
|  | Strongly agree | 41% |  |  |  |  |  |
|  |  |  |  |  |  |  |  |
| Serious consequences if infected ^6^ | Strongly disagree | 25% | 1.23 (1.1, 1.38) | <0.001 | 1.24 (1.09, 1.4) | 0.001 |  |
|  | Somewhat disagree | 33% |  |  |  |  |  |
|  | Neither agree nor disagree | 38% |  |  |  |  |  |
|  | Somewhat agree | 39% |  |  |  |  |  |
|  | Strongly agree | 50% |  |  |  |  |  |
|  |  |  |  |  |  |  |  |
| Have you heard about the use of Paxlovid medication to reduce COVID-19 severity? | | 52% | 0.88 (0.7, 1.11) | 0.277 | 0.87 (0.68, 1.12) | 0.290 |  |
| Note: |  |  |  |  |  |  |  |
| ^1^ *Percentages shown are row percentages, and are based on all respondents to the 39^th^ survey wave on Paxlovid who had valid responses to that variable, and includes 1432 observations unless otherwise mentioned*  *^2^ Crude Odds Ratios and Adjusted Odds Ratios include only 1240 observations (excludes 2 respondents with missing household income data and another 190 respondents who did not have previous survey responses on trust in government and healthcare providers), with Adjusted Odds Ratios based on multivariable analyses adjusting for all variables in the above table* | | | | | | |  |
| ^3^ Excludes 2 respondents who did not disclose household income. | | | | | | |  |
| ^4^ Variables are in binary format where the reference category is those who did not cite that information source. | | | | | | |  |
| ^5^ Excludes 190 respondents who did not have previous survey responses on trust in government and healthcare providers.  ^6^ These variables are analysed on an ordinal scale format. | | | | | | |  |
|  | | | | | | |  |
|  |  |  |  |  |  |  |  |

Table S6 – Multivariable regression analysis showing factors associated with concern about the side-effects of Paxlovid

| **Category** | | **% of respondents^1^** | **Crude Odds Ratio (CI)^2^** | **p-value** | **Adjusted Odds Ratio (CI)^2^** | **p-value** |  |
| --- | --- | --- | --- | --- | --- | --- | --- |
| Recruitment method | Door to door | 54 (54%) | reference |  | reference |  |  |
|  | Referral | 379 (66%) | 1.62 (1.06, 2.49) | 0.027 | 1.62 (1.02, 2.56) | 0.039 |  |
|  | Other cohort | 353 (63%) | 1.44 (0.94, 2.21) | 0.096 | 1.48 (0.93, 2.35) | 0.096 |  |
|  |  |  |  |  |  |  |  |
| Age group | Below 30 | 102 (61%) | reference |  | reference |  |  |
|  | 30 - 39 | 152 (66%) | 1.24 (0.82, 1.88) | 0.313 | 1.18 (0.75, 1.85) | 0.465 |  |
|  | 40 - 49 | 156 (65%) | 1.15 (0.76, 1.73) | 0.499 | 1.14 (0.72, 1.82) | 0.575 |  |
|  | 50 - 59 | 174 (65%) | 1.15 (0.77, 1.71) | 0.496 | 1.21 (0.76, 1.93) | 0.422 |  |
|  | 60 and above | 202 (60%) | 0.95 (0.65, 1.4) | 0.805 | 1.01 (0.62, 1.63) | 0.969 |  |
|  |  |  |  |  |  |  |  |
| Gender | Male | 290 (58%) | reference |  | reference |  |  |
|  | Female | 496 (67%) | 1.5 (1.19, 1.89) | 0.001 | 1.37 (1.07, 1.76) | 0.012 |  |
|  |  |  |  |  |  |  |  |
| Race | Chinese | 704 (64%) | reference |  | reference |  |  |
|  | Malay | 19 (70%) | 1.35 (0.58, 3.1) | 0.486 | 1.4 (0.58, 3.34) | 0.455 |  |
|  | Indian | 45 (57%) | 0.75 (0.47, 1.19) | 0.223 | 0.79 (0.49, 1.29) | 0.348 |  |
|  | Others | 18 (58%) | 0.78 (0.38, 1.62) | 0.512 | 0.89 (0.42, 1.89) | 0.766 |  |
|  |  |  |  |  |  |  |  |
| Highest education | 'O' / 'N' level & below | 114 (64%) | reference |  | reference |  |  |
|  | 'A' level / Polytechnic diploma | 210 (62%) | 0.92 (0.63, 1.34) | 0.669 | 0.88 (0.59, 1.32) | 0.537 |  |
|  | University / Post-graduate | 462 (64%) | 0.99 (0.7, 1.39) | 0.954 | 0.99 (0.67, 1.46) | 0.963 |  |
|  |  |  |  |  |  |  |  |
| Housing type | Privately owned property | 111 (62%) | reference |  | reference |  |  |
|  | Publicly owned flat with ≤ 3 rooms | 457 (64%) | 0.97 (0.66, 1.4) | 0.853 | 0.93 (0.61, 1.42) | 0.743 |  |
|  | Publicly owned flat with 4–5 rooms | 218 (63%) | 1.02 (0.78, 1.34) | 0.862 | 1 (0.74, 1.33) | 0.978 |  |
|  |  |  |  |  |  |  |  |
| Monthly household income (SGD)^3^ | ≤ 4999 | 269 (64%) | reference |  | reference |  |  |
|  | 5000–8999 | 216 (64%) | 1.02 (0.75, 1.37) | 0.911 | 1 (0.73, 1.38) | 0.984 |  |
|  | ≥ 9000 | 301 (62%) | 0.93 (0.71, 1.23) | 0.624 | 0.9 (0.66, 1.22) | 0.504 |  |
|  |  |  |  |  |  |  |  |
| *Preexisting conditions:* |  |  |  |  |  |  |  |
| Diabetes | No | 24 (42%) | reference |  | reference |  |  |
|  | Yes | 33 (58%) | 0.79 (0.46, 1.35) | 0.379 | 0.92 (0.51, 1.67) | 0.786 |  |
|  |  | 56 (45%) | reference |  | reference |  |  |
| Hypertension | No | 68 (55%) | 0.67 (0.46, 0.98) | 0.038 | 0.72 (0.47, 1.1) | 0.129 |  |
|  | Yes |  |  |  |  |  |  |
|  |  |  |  |  |  |  |  |
| *Where do you usually get information about outbreaks of infectious diseases in Singapore? ^4^* | |  |  |  |  |  |  |
| TV |  | 502 (63%) | 0.94 (0.74, 1.2) | 0.637 | 0.94 (0.71, 1.23) | 0.653 |  |
| Radio |  | 223 (65%) | 1.1 (0.85, 1.43) | 0.462 | 1.13 (0.84, 1.51) | 0.418 |  |
| Print media (e.g. newspapers / fliers / notice boards / posters / banners / bus-stop boards) | | 389 (63%) | 0.96 (0.76, 1.21) | 0.747 | 1.01 (0.78, 1.3) | 0.964 |  |
| Family / relatives |  | 374 (67%) | 1.28 (1.02, 1.62) | 0.036 | 1.28 (0.94, 1.75) | 0.117 |  |
| Friends / colleagues |  | 456 (65%) | 1.16 (0.92, 1.46) | 0.217 | 0.95 (0.7, 1.29) | 0.742 |  |
| Social media (e.g. Facebook / Instagram / Twitter / Straits Times / Facebook Page) | | 613 (64%) | 1.09 (0.83, 1.44) | 0.527 | 1.03 (0.77, 1.39) | 0.831 |  |
| Websites (e.g. Google / Yahoo / MSN / Straits Times Online / MOH) | | 530 (63%) | 0.95 (0.74, 1.22) | 0.697 | 0.88 (0.68, 1.15) | 0.343 |  |
|  |  |  |  |  |  |  |  |
| Trust in healthcare providers ^5,6^ | Strongly disagree | 6 (86%) | 0.7 (0.57, 0.87) | 0.001 | 0.78 (0.6, 1.02) | 0.071 |  |
|  | Disagree | 18 (72%) |  |  |  |  |  |
|  | Agree | 458 (67%) |  |  |  |  |  |
|  | Strongly agree | 304 (58%) |  |  |  |  |  |
|  |  |  |  |  |  |  |  |
| Trust in government communications ^5,6^ | Strongly disagree | 24 (73%) | 0.83 (0.69, 0.98) | 0.033 | 0.96 (0.77, 1.19) | 0.715 |  |
|  | Disagree | 66 (62%) |  |  |  |  |  |
|  | Agree | 501 (66%) |  |  |  |  |  |
|  | Strongly agree | 195 (58%) |  |  |  |  |  |
|  |  |  |  |  |  |  |  |
| Likely to get COVID-19 infection ^6^ | Strongly disagree | 33 (77%) | 1.01 (0.9, 1.13) | 0.867 | 0.95 (0.84, 1.08) | 0.459 |  |
|  | Somewhat disagree | 62 (55%) |  |  |  |  |  |
|  | Neither agree nor disagree | 258 (63%) |  |  |  |  |  |
|  | Somewhat agree | 287 (66%) |  |  |  |  |  |
|  | Strongly agree | 146 (62%) |  |  |  |  |  |
|  |  |  |  |  |  |  |  |
| Serious consequences if infected ^6^ | Strongly disagree | 39 (61%) | 1.19 (1.07, 1.33) | 0.002 | 1.2 (1.06, 1.35) | 0.004 |  |
|  | Somewhat disagree | 136 (55%) |  |  |  |  |  |
|  | Neither agree nor disagree | 258 (62%) |  |  |  |  |  |
|  | Somewhat agree | 267 (70%) |  |  |  |  |  |
|  | Strongly agree | 86 (66%) |  |  |  |  |  |
|  |  |  |  |  |  |  |  |
| Have you heard about the use of Paxlovid medication to reduce COVID-19 severity? | | 434 (65%) | 1.13 (0.9, 1.42) | 0.305 | 1.2 (0.94, 1.54) | 0.142 |  |
| Note: |  |  |  |  |  |  |  |
| ^1^ *Percentages shown are row percentages, and are based on all respondents to the 39^th^ survey wave on Paxlovid who had valid responses to that variable, and includes 1432 observations unless otherwise mentioned*  *^2^ Crude Odds Ratios and Adjusted Odds Ratios include only 1240 observations (excludes 2 respondents with missing household income data and another 190 respondents who did not have previous survey responses on trust in government and healthcare providers), with Adjusted Odds Ratios based on multivariable analyses adjusting for all variables in the above table* | | | | | | |  |
| ^3^ Excludes 2 respondents who did not disclose household income. | | | | | | |  |
| ^4^ Variables are in binary format where the reference category is those who did not cite that information source. | | | | | | |  |
| ^5^ Excludes 190 respondents who did not have previous survey responses on trust in government and healthcare providers.  ^6^ These variables are analysed on an ordinal scale format. | | | | | | |  |
|  | | | | | | |  |
|  |  |  |  |  |  |  |  |

Table S7 – Multivariable regression analysis showing factors associated with concern of the cost of Paxlovid

| **Category** | | **% of respondents^1^** | **Crude Odds Ratio (CI)^2^** | **p-value** | **Adjusted Odds Ratio (CI)^2^** | **p-value** |  |
| --- | --- | --- | --- | --- | --- | --- | --- |
| Recruitment method | Door to door | 32 (32%) | reference |  | reference |  |  |
|  | Referral | 166 (29%) | 0.86 (0.54, 1.35) | 0.506 | 1 (0.61, 1.62) | 0.989 |  |
|  | Other cohort | 162 (29%) | 0.86 (0.54, 1.36) | 0.521 | 1.21 (0.74, 1.98) | 0.450 |  |
|  |  |  |  |  |  |  |  |
| Age group | Below 30 | 54 (33%) | reference |  | reference |  |  |
|  | 30 - 39 | 82 (36%) | 1.16 (0.76, 1.77) | 0.499 | 1.03 (0.65, 1.63) | 0.906 |  |
|  | 40 - 49 | 69 (29%) | 0.83 (0.54, 1.28) | 0.4 | 0.69 (0.42, 1.13) | 0.144 |  |
|  | 50 - 59 | 73 (27%) | 0.77 (0.51, 1.18) | 0.23 | 0.61 (0.37, 1) | 0.052 |  |
|  | 60 and above | 82 (24%) | 0.67 (0.45, 1.01) | 0.057 | 0.55 (0.33, 0.91) | 0.022 |  |
|  |  |  |  |  |  |  |  |
| Gender | Male | 161 (32%) | reference |  | reference |  |  |
|  | Female | 199 (27%) | 0.78 (0.61, 1) | 0.052 | 0.75 (0.58, 0.98) | 0.035 |  |
|  |  |  |  |  |  |  |  |
| Race | Chinese | 315 (29%) | reference |  | reference |  |  |
|  | Malay | 8 (30%) | 1.05 (0.46, 2.43) | 0.903 | 0.82 (0.34, 1.99) | 0.666 |  |
|  | Indian | 26 (33%) | 1.23 (0.75, 2) | 0.41 | 1.14 (0.68, 1.9) | 0.618 |  |
|  | Others | 11 (35%) | 1.38 (0.65, 2.9) | 0.403 | 1.33 (0.62, 2.88) | 0.463 |  |
|  |  |  |  |  |  |  |  |
| Highest education | 'O' / 'N' level & below | 55 (31%) | reference |  | reference |  |  |
|  | 'A' level / Polytechnic diploma | 102 (30%) | 0.97 (0.65, 1.43) | 0.866 | 0.87 (0.57, 1.33) | 0.509 |  |
|  | University / Post-graduate | 203 (28%) | 0.87 (0.61, 1.25) | 0.45 | 0.78 (0.52, 1.18) | 0.244 |  |
|  |  |  |  |  |  |  |  |
| Housing type | Privately owned property | 54 (30%) | reference |  | reference |  |  |
|  | Publicly owned flat with ≤ 3 rooms | 232 (32%) | 1.59 (1.06, 2.4) | 0.026 | 1.56 (0.99, 2.45) | 0.056 |  |
|  | Publicly owned flat with 4–5 rooms | 74 (21%) | 1.75 (1.3, 2.37) | <0.001 | 1.58 (1.14, 2.19) | 0.006 |  |
|  |  |  |  |  |  |  |  |
| Monthly household income (SGD)^3^ | ≤ 4999 | 125 (30%) | reference |  | reference |  |  |
|  | 5000–8999 | 106 (32%) | 1.09 (0.8, 1.49) | 0.582 | 1.02 (0.73, 1.42) | 0.925 |  |
|  | ≥ 9000 | 129 (27%) | 0.86 (0.65, 1.15) | 0.32 | 0.87 (0.62, 1.21) | 0.401 |  |
|  |  |  |  |  |  |  |  |
| *Preexisting conditions:* |  |  |  |  |  |  |  |
| Diabetes | No | 40 (70%) | reference |  | reference |  |  |
|  | Yes | 17 (30%) | 1.04 (0.58, 1.86) | 0.893 | 1.34 (0.7, 2.56) | 0.380 |  |
|  |  | 93 (75%) | reference |  | reference |  |  |
| Hypertension | No | 31 (25%) | 0.8 (0.52, 1.22) | 0.298 | 0.75 (0.46, 1.22) | 0.240 |  |
|  | Yes |  |  |  |  |  |  |
|  |  |  |  |  |  |  |  |
| *Where do you usually get information about outbreaks of infectious diseases in Singapore? ^4^* | |  |  |  |  |  |  |
| TV |  | 235 (29%) | 1.06 (0.82, 1.37) | 0.664 | 1.1 (0.82, 1.48) | 0.518 |  |
| Radio |  | 106 (31%) | 1.13 (0.86, 1.48) | 0.369 | 1.28 (0.94, 1.74) | 0.115 |  |
| Print media (e.g. newspapers / fliers / notice boards / posters / banners / bus-stop boards) | | 180 (29%) | 1.01 (0.79, 1.29) | 0.942 | 1.04 (0.79, 1.37) | 0.780 |  |
| Family / relatives |  | 156 (28%) | 0.89 (0.7, 1.14) | 0.368 | 0.86 (0.61, 1.2) | 0.380 |  |
| Friends / colleagues |  | 196 (28%) | 0.88 (0.69, 1.13) | 0.307 | 0.96 (0.69, 1.33) | 0.793 |  |
| Social media (e.g. Facebook / Instagram / Twitter / Straits Times / Facebook Page) | | 271 (28%) | 0.84 (0.63, 1.13) | 0.249 | 0.72 (0.53, 0.99) | 0.044 |  |
| Websites (e.g. Google / Yahoo / MSN / Straits Times Online / MOH) | | 257 (31%) | 1.26 (0.97, 1.65) | 0.086 | 1.35 (1.01, 1.8) | 0.041 |  |
|  |  |  |  |  |  |  |  |
| Trust in healthcare providers ^5,6^ | Strongly disagree | 1 (14%) | 0.94 (0.76, 1.17) | 0.603 | 0.96 (0.73, 1.27) | 0.800 |  |
|  | Disagree | 8 (32%) |  |  |  |  |  |
|  | Agree | 206 (30%) |  |  |  |  |  |
|  | Strongly agree | 145 (28%) |  |  |  |  |  |
|  |  |  |  |  |  |  |  |
| Trust in government communications ^5,6^ | Strongly disagree | 9 (27%) | 0.95 (0.79, 1.14) | 0.562 | 0.98 (0.77, 1.23) | 0.834 |  |
|  | Disagree | 30 (28%) |  |  |  |  |  |
|  | Agree | 231 (30%) |  |  |  |  |  |
|  | Strongly agree | 90 (27%) |  |  |  |  |  |
|  |  |  |  |  |  |  |  |
| Likely to get COVID-19 infection ^6^ | Strongly disagree | 12 (28%) | 1.19 (1.05, 1.35) | 0.007 | 1.1 (0.96, 1.27) | 0.168 |  |
|  | Somewhat disagree | 27 (24%) |  |  |  |  |  |
|  | Neither agree nor disagree | 109 (26%) |  |  |  |  |  |
|  | Somewhat agree | 124 (28%) |  |  |  |  |  |
|  | Strongly agree | 88 (38%) |  |  |  |  |  |
|  |  |  |  |  |  |  |  |
| Serious consequences if infected ^6^ | Strongly disagree | 14 (22%) | 1.27 (1.12, 1.43) | <0.001 | 1.22 (1.07, 1.39) | 0.004 |  |
|  | Somewhat disagree | 59 (24%) |  |  |  |  |  |
|  | Neither agree nor disagree | 103 (25%) |  |  |  |  |  |
|  | Somewhat agree | 139 (36%) |  |  |  |  |  |
|  | Strongly agree | 45 (35%) |  |  |  |  |  |
|  |  |  |  |  |  |  |  |
| Have you heard about the use of Paxlovid medication to reduce COVID-19 severity? | | 189 (28%) | 0.91 (0.71, 1.17) | 0.466 | 0.92 (0.71, 1.2) | 0.560 |  |
| Note: |  |  |  |  |  |  |  |
| ^1^ *Percentages shown are row percentages, and are based on all respondents to the 39^th^ survey wave on Paxlovid who had valid responses to that variable, and includes 1432 observations unless otherwise mentioned*  *^2^ Crude Odds Ratios and Adjusted Odds Ratios include only 1240 observations (excludes 2 respondents with missing household income data and another 190 respondents who did not have previous survey responses on trust in government and healthcare providers), with Adjusted Odds Ratios based on multivariable analyses adjusting for all variables in the above table* | | | | | | |  |
| ^3^ Excludes 2 respondents who did not disclose household income. | | | | | | |  |
| ^4^ Variables are in binary format where the reference category is those who did not cite that information source. | | | | | | |  |
| ^5^ Excludes 190 respondents who did not have previous survey responses on trust in government and healthcare providers.  ^6^ These variables are analysed on an ordinal scale format. | | | | | | |  |
|  | | | | | | |  |
|  |  |  |  |  |  |  |  |

Table S8 – Multivariable regression analysis showing factors associated with concern of the ineffectiveness of Paxlovid

| **Category** | | **% of respondents^1^** | **Crude Odds Ratio (CI)^2^** | **p-value** | **Adjusted Odds Ratio (CI)^2^** | **p-value** |  |
| --- | --- | --- | --- | --- | --- | --- | --- |
| Recruitment method | Door to door | 5 (5%) | reference |  | reference |  |  |
|  | Referral | 53 (9%) | 1.92 (0.75, 4.92) | 0.176 | 1.63 (0.6, 4.38) | 0.337 |  |
|  | Other cohort | 42 (7%) | 1.53 (0.59, 3.98) | 0.378 | 1.49 (0.54, 4.08) | 0.441 |  |
|  |  |  |  |  |  |  |  |
| Age group | Below 30 | 12 (7%) | reference |  | reference |  |  |
|  | 30 - 39 | 25 (11%) | 1.57 (0.77, 3.23) | 0.217 | 1.61 (0.73, 3.54) | 0.234 |  |
|  | 40 - 49 | 15 (6%) | 0.85 (0.39, 1.87) | 0.689 | 0.93 (0.38, 2.25) | 0.870 |  |
|  | 50 - 59 |  | 1.14 (0.55, 2.38) | 0.72 | 1.33 (0.56, 3.13) | 0.515 |  |
|  | 60 and above | 26 (8%) | 1.08 (0.53, 2.2) | 0.832 | 1.28 (0.53, 3.08) | 0.579 |  |
|  |  |  |  |  |  |  |  |
| Gender | Male | 36 (7%) | reference |  | reference |  |  |
|  | Female | 64 (9%) | 1.23 (0.8, 1.88) | 0.341 | 1.21 (0.75, 1.93) | 0.434 |  |
|  |  |  |  |  |  |  |  |
| Race | Chinese | 92 (8%) | reference |  | reference |  |  |
|  | Malay | 1 (4%) | 0.42 (0.06, 3.15) | 0.401 | 0.29 (0.04, 2.29) | 0.240 |  |
|  | Indian | 5 (6%) | 0.74 (0.29, 1.88) | 0.531 | 0.67 (0.25, 1.78) | 0.422 |  |
|  | Others | 2 (6%) | 0.76 (0.18, 3.23) | 0.708 | 0.78 (0.17, 3.54) | 0.747 |  |
|  |  |  |  |  |  |  |  |
| Highest education | 'O' / 'N' level & below | 15 (8%) | reference |  | reference |  |  |
|  | 'A' level / Polytechnic diploma | 35 (10%) | 1.26 (0.67, 2.37) | 0.482 | 1.22 (0.61, 2.47) | 0.571 |  |
|  | University / Post-graduate | 50 (7%) | 0.81 (0.44, 1.47) | 0.483 | 0.91 (0.45, 1.84) | 0.800 |  |
|  |  |  |  |  |  |  |  |
| Housing type | Privately owned property | 13 (7%) | reference |  | reference |  |  |
|  | Publicly owned flat with ≤ 3 rooms | 66 (9%) | 1.22 (0.59, 2.49) | 0.593 | 0.99 (0.44, 2.23) | 0.990 |  |
|  | Publicly owned flat with 4–5 rooms | 21 (6%) | 1.56 (0.94, 2.6) | 0.085 | 1.43 (0.82, 2.48) | 0.203 |  |
|  |  |  |  |  |  |  |  |
| Monthly household income (SGD)^3^ | ≤ 4999 | 41 (10%) | reference |  | reference |  |  |
|  | 5000–8999 | 31 (9%) | 0.94 (0.58, 1.54) | 0.811 | 0.9 (0.53, 1.54) | 0.702 |  |
|  | ≥ 9000 | 28 (6%) | 0.57 (0.35, 0.94) | 0.028 | 0.59 (0.34, 1.04) | 0.068 |  |
|  |  |  |  |  |  |  |  |
| *Preexisting conditions:* |  |  |  |  |  |  |  |
| Diabetes | No | 52 (91%) | reference |  | reference |  |  |
|  | Yes | 5 (9%) | 1.1 (0.43, 2.82) | 0.841 | 1.38 (0.49, 3.91) | 0.547 |  |
|  |  | 114 (92%) | reference |  | reference |  |  |
| Hypertension | No | 10 (8%) | 1 (0.51, 1.98) | 1 | 0.88 (0.39, 1.95) | 0.745 |  |
|  | Yes |  |  |  |  |  |  |
|  |  |  |  |  |  |  |  |
| *Where do you usually get information about outbreaks of infectious diseases in Singapore? ^4^* | |  |  |  |  |  |  |
| TV |  | 64 (8%) | 0.98 (0.64, 1.51) | 0.938 | 0.98 (0.6, 1.62) | 0.946 |  |
| Radio |  | 33 (10%) | 1.32 (0.85, 2.04) | 0.214 | 1.42 (0.86, 2.33) | 0.170 |  |
| Print media (e.g. newspapers / fliers / notice boards / posters / banners / bus-stop boards) | | 46 (7%) | 0.85 (0.56, 1.27) | 0.424 | 0.86 (0.54, 1.38) | 0.541 |  |
| Family / relatives |  | 53 (9%) | 1.4 (0.93, 2.11) | 0.109 | 1.62 (0.9, 2.88) | 0.105 |  |
| Friends / colleagues |  | 56 (8%) | 0.97 (0.64, 1.46) | 0.884 | 0.69 (0.38, 1.22) | 0.203 |  |
| Social media (e.g. Facebook / Instagram / Twitter / Straits Times / Facebook Page) | | 84 (9%) | 1.58 (0.91, 2.75) | 0.103 | 1.56 (0.86, 2.83) | 0.143 |  |
| Websites (e.g. Google / Yahoo / MSN / Straits Times Online / MOH) | | 65 (8%) | 0.87 (0.57, 1.34) | 0.529 | 0.91 (0.57, 1.45) | 0.692 |  |
|  |  |  |  |  |  |  |  |
| Trust in healthcare providers ^5,6^ | Strongly disagree | 4 (57%) | 0.44 (0.31, 0.63) | <0.001 | 0.84 (0.54, 1.3) | 0.439 |  |
|  | Disagree | 8 (32%) |  |  |  |  |  |
|  | Agree | 58 (8%) |  |  |  |  |  |
|  | Strongly agree | 30 (6%) |  |  |  |  |  |
|  |  |  |  |  |  |  |  |
| Trust in government communications ^5,6^ | Strongly disagree | 10 (30%) | 0.43 (0.32, 0.56) | <0.001 | 0.46 (0.33, 0.64) | <0.001 |  |
|  | Disagree | 23 (22%) |  |  |  |  |  |
|  | Agree | 51 (7%) |  |  |  |  |  |
|  | Strongly agree | 16 (5%) |  |  |  |  |  |
|  |  |  |  |  |  |  |  |
| Likely to get COVID-19 infection ^6^ | Strongly disagree | 5 (12%) | 0.92 (0.75, 1.12) | 0.406 | 0.85 (0.67, 1.08) | 0.188 |  |
|  | Somewhat disagree | 9 (8%) |  |  |  |  |  |
|  | Neither agree nor disagree | 39 (9%) |  |  |  |  |  |
|  | Somewhat agree | 26 (6%) |  |  |  |  |  |
|  | Strongly agree | 21 (9%) |  |  |  |  |  |
|  |  |  |  |  |  |  |  |
| Serious consequences if infected ^6^ | Strongly disagree | 10 (16%) | 1.18 (0.97, 1.44) | 0.102 | 1.2 (0.95, 1.51) | 0.130 |  |
|  | Somewhat disagree | 10 (4%) |  |  |  |  |  |
|  | Neither agree nor disagree | 26 (6%) |  |  |  |  |  |
|  | Somewhat agree | 40 (10%) |  |  |  |  |  |
|  | Strongly agree | 14 (11%) |  |  |  |  |  |
|  |  |  |  |  |  |  |  |
| Have you heard about the use of Paxlovid medication to reduce COVID-19 severity? | | 45 (7%) | 0.67 (0.45, 1.01) | 0.058 | 0.67 (0.43, 1.04) | 0.077 |  |
| Note: |  |  |  |  |  |  |  |
| ^1^ *Percentages shown are row percentages, and are based on all respondents to the 39^th^ survey wave on Paxlovid who had valid responses to that variable, and includes 1432 observations unless otherwise mentioned*  *^2^ Crude Odds Ratios and Adjusted Odds Ratios include only 1240 observations (excludes 2 respondents with missing household income data and another 190 respondents who did not have previous survey responses on trust in government and healthcare providers), with Adjusted Odds Ratios based on multivariable analyses adjusting for all variables in the above table* | | | | | | |  |
| ^3^ Excludes 2 respondents who did not disclose household income. | | | | | | |  |
| ^4^ Variables are in binary format where the reference category is those who did not cite that information source. | | | | | | |  |
| ^5^ Excludes 190 respondents who did not have previous survey responses on trust in government and healthcare providers.  ^6^ These variables are analysed on an ordinal scale format. | | | | | | |  |
|  | | | | | | |  |
|  |  |  |  |  |  |  |  |

Table S9 – Multivariable regression analysis showing factors associated with COVID-19 being mild disease

| **Category** | | **% of respondents^1^** | **Crude Odds Ratio (CI)^2^** | **p-value** | **Adjusted Odds Ratio (CI)^2^** | **p-value** |  |
| --- | --- | --- | --- | --- | --- | --- | --- |
| Recruitment method | Door to door | 19 (19%) | reference |  | reference |  |  |
|  | Referral | 151 (26%) | 1.51 (0.88, 2.57) | 0.131 | 1.72 (0.95, 3.11) | 0.074 |  |
|  | Other cohort | 142 (25%) | 1.44 (0.84, 2.46) | 0.18 | 1.39 (0.77, 2.54) | 0.277 |  |
|  |  |  |  |  |  |  |  |
| Age group | Below 30 | 41 (25%) | reference |  | reference |  |  |
|  | 30 - 39 | 60 (26%) | 1.08 (0.68, 1.71) | 0.736 | 1.24 (0.74, 2.09) | 0.415 |  |
|  | 40 - 49 | 56 (23%) | 0.92 (0.58, 1.47) | 0.734 | 1.13 (0.66, 1.96) | 0.652 |  |
|  | 50 - 59 | 58 (0%) | 0.84 (0.53, 1.32) | 0.449 | 1.15 (0.67, 1.99) | 0.613 |  |
|  | 60 and above | 97 (29%) | 1.24 (0.81, 1.9) | 0.316 | 1.75 (1.01, 3.04) | 0.048 |  |
|  |  |  |  |  |  |  |  |
| Gender | Male | 146 (29%) | reference |  | reference |  |  |
|  | Female | 166 (22%) | 0.71 (0.55, 0.92) | 0.009 | 0.63 (0.47, 0.84) | 0.002 |  |
|  |  |  |  |  |  |  |  |
| Race | Chinese | 280 (25%) | reference |  | reference |  |  |
|  | Malay | 3 (11%) | 0.37 (0.11, 1.23) | 0.104 | 0.5 (0.14, 1.75) | 0.279 |  |
|  | Indian | 16 (20%) | 0.75 (0.42, 1.31) | 0.311 | 0.78 (0.42, 1.44) | 0.423 |  |
|  | Others | 13 (42%) | 2.12 (1.03, 4.39) | 0.042 | 2.35 (1.08, 5.12) | 0.032 |  |
|  |  |  |  |  |  |  |  |
| Highest education | 'O' / 'N' level & below | 46 (26%) | reference |  | reference |  |  |
|  | 'A' level / Polytechnic diploma | 72 (21%) | 0.78 (0.51, 1.19) | 0.244 | 0.7 (0.43, 1.12) | 0.135 |  |
|  | University / Post-graduate | 194 (27%) | 1.05 (0.72, 1.53) | 0.797 | 0.86 (0.55, 1.34) | 0.498 |  |
|  |  |  |  |  |  |  |  |
| Housing type | Privately owned property | 42 (24%) | reference |  | reference |  |  |
|  | Publicly owned flat with ≤ 3 rooms | 175 (24%) | 0.81 (0.53, 1.24) | 0.332 | 1.01 (0.63, 1.64) | 0.954 |  |
|  | Publicly owned flat with 4–5 rooms | 95 (28%) | 0.85 (0.63, 1.14) | 0.273 | 0.98 (0.7, 1.36) | 0.897 |  |
|  |  |  |  |  |  |  |  |
| Monthly household income (SGD)^3^ | ≤ 4999 | 89 (21%) | reference |  | reference |  |  |
|  | 5000–8999 | 86 (26%) | 1.28 (0.91, 1.8) | 0.149 | 1.23 (0.84, 1.79) | 0.290 |  |
|  | ≥ 9000 | 137 (28%) | 1.48 (1.09, 2.01) | 0.013 | 1.38 (0.96, 1.97) | 0.082 |  |
|  |  |  |  |  |  |  |  |
| *Preexisting conditions:* |  |  |  |  |  |  |  |
| Diabetes | No | 51 (89%) | reference |  | reference |  |  |
|  | Yes | 6 (11%) | 0.34 (0.14, 0.79) | 0.013 | 0.38 (0.15, 0.97) | 0.043 |  |
|  |  | 105 (85%) | reference |  | reference |  |  |
| Hypertension | No | 19 (15%) | 0.51 (0.31, 0.84) | 0.009 | 0.52 (0.29, 0.91) | 0.022 |  |
|  | Yes |  |  |  |  |  |  |
|  |  |  |  |  |  |  |  |
| *Where do you usually get information about outbreaks of infectious diseases in Singapore? ^4^* | |  |  |  |  |  |  |
| TV |  | 208 (26%) | 1.15 (0.87, 1.5) | 0.325 | 1.19 (0.86, 1.65) | 0.281 |  |
| Radio |  | 92 (27%) | 1.13 (0.85, 1.5) | 0.405 | 1.02 (0.73, 1.43) | 0.902 |  |
| Print media (e.g. newspapers / fliers / notice boards / posters / banners / bus-stop boards) | | 153 (25%) | 0.96 (0.74, 1.24) | 0.744 | 0.83 (0.62, 1.11) | 0.211 |  |
| Family / relatives |  | 154 (27%) | 1.24 (0.96, 1.61) | 0.098 | 1.02 (0.71, 1.46) | 0.903 |  |
| Friends / colleagues |  | 200 (28%) | 1.51 (1.16, 1.97) | 0.002 | 1.49 (1.03, 2.13) | 0.032 |  |
| Social media (e.g. Facebook / Instagram / Twitter / Straits Times / Facebook Page) | | 249 (26%) | 1.21 (0.88, 1.65) | 0.244 | 1.12 (0.79, 1.59) | 0.532 |  |
| Websites (e.g. Google / Yahoo / MSN / Straits Times Online / MOH) | | 218 (26%) | 1.14 (0.86, 1.5) | 0.371 | 1.06 (0.78, 1.44) | 0.723 |  |
|  |  |  |  |  |  |  |  |
| Trust in healthcare providers ^5,6^ | Strongly disagree | 1 (14%) | 1.2 (0.95, 1.51) | 0.129 | 1.01 (0.74, 1.37) | 0.946 |  |
|  | Disagree | 2 (8%) |  |  |  |  |  |
|  | Agree | 171 (25%) |  |  |  |  |  |
|  | Strongly agree | 138 (27%) |  |  |  |  |  |
|  |  |  |  |  |  |  |  |
| Trust in government communications ^5,6^ | Strongly disagree | 7 (21%) | 1.07 (0.88, 1.3) | 0.479 | 1.03 (0.8, 1.32) | 0.818 |  |
|  | Disagree | 28 (26%) |  |  |  |  |  |
|  | Agree | 186 (24%) |  |  |  |  |  |
|  | Strongly agree | 91 (27%) |  |  |  |  |  |
|  |  |  |  |  |  |  |  |
| Likely to get COVID-19 infection ^6^ | Strongly disagree | 13 (30%) | 0.89 (0.79, 1.01) | 0.081 | 1.09 (0.94, 1.26) | 0.266 |  |
|  | Somewhat disagree | 33 (29%) |  |  |  |  |  |
|  | Neither agree nor disagree | 107 (26%) |  |  |  |  |  |
|  | Somewhat agree | 108 (25%) |  |  |  |  |  |
|  | Strongly agree | 51 (22%) |  |  |  |  |  |
|  |  |  |  |  |  |  |  |
| Serious consequences if infected ^6^ | Strongly disagree | 34 (53%) | 0.55 (0.48, 0.63) | <0.001 | 0.54 (0.46, 0.62) | <0.001 |  |
|  | Somewhat disagree | 103 (42%) |  |  |  |  |  |
|  | Neither agree nor disagree | 100 (24%) |  |  |  |  |  |
|  | Somewhat agree | 56 (15%) |  |  |  |  |  |
|  | Strongly agree | 19 (15%) |  |  |  |  |  |
|  |  |  |  |  |  |  |  |
| Have you heard about the use of Paxlovid medication to reduce COVID-19 severity? | | 170 (25%) | 1.02 (0.79, 1.32) | 88% | 0.96 (0.72, 1.27) | 0.757 |  |
| Note: |  |  |  |  |  |  |  |
| ^1^ *Percentages shown are row percentages, and are based on all respondents to the 39^th^ survey wave on Paxlovid who had valid responses to that variable, and includes 1432 observations unless otherwise mentioned*  *^2^ Crude Odds Ratios and Adjusted Odds Ratios include only 1240 observations (excludes 2 respondents with missing household income data and another 190 respondents who did not have previous survey responses on trust in government and healthcare providers), with Adjusted Odds Ratios based on multivariable analyses adjusting for all variables in the above table* | | | | | | |  |
| ^3^ Excludes 2 respondents who did not disclose household income. | | | | | | |  |
| ^4^ Variables are in binary format where the reference category is those who did not cite that information source. | | | | | | |  |
| ^5^ Excludes 190 respondents who did not have previous survey responses on trust in government and healthcare providers.  ^6^ These variables are analysed on an ordinal scale format. | | | | | | |  |
|  | | | | | | |  |
|  |  |  |  |  |  |  |  |
